# Laser Therapy for the Treatment of Actinic Cheilitis: A Systematic Review

**DOI:** 10.3390/ijerph19084593

**Published:** 2022-04-11

**Authors:** Angela Ayen-Rodriguez, Maria Jose Naranjo-Diaz, Ricardo Ruiz-Villaverde

**Affiliations:** Dermatology Department, Hospital Universitario San Cecilio, 18200 Granada, Spain; mjnaranjodiaz@hotmail.com (M.J.N.-D.); ismenios2005@gmail.com (R.R.-V.)

**Keywords:** actinic cheilitis, laser therapy, carbon dioxide laser, Erbium:YAG laser, 1927-nm thulium laser, keratosis, actinic, oral potentially malignant disorders, lip cancer, non-melanoma skin cancer, systematic review

## Abstract

Actinic cheilitis (AC) is a chronic inflammation of the lip considered an oral, potentially malignant disorder associated with an increased risk of lip squamous cell carcinoma (SCC) development. Controversies surrounding current therapeutic modalities of AC are under debate, and the implications of laser treatment have not been specifically investigated through a systematic review design. The present study aims to evaluate the degree of evidence of laser for the treatment of AC in terms of efficacy and safety. We searched for primary-level studies published before January 2022 through MEDLINE/PubMed, Embase, Web of Science, Scopus and CENTRAL, with no limitation in publication language or date. We evaluated the methodological quality and risk of bias of the studies included using the updated Cochrane Collaboration’s tool for assessing risk of bias (RoB-2). Twenty studies (512 patients) met our eligibility criteria. Laser therapy showed a complete clearance of AC in 92.5% patients, with a maximum recurrence rate of 21.43%, and a very low frequency of malignant transformation to SCC (detected in only 3/20 studies analyzed). In addition, cosmetic outcomes and patient satisfaction were described as excellent. In conclusion, our findings indicate that laser therapy is a high efficacy approach to AC.

## 1. Introduction

**Figure 1:**
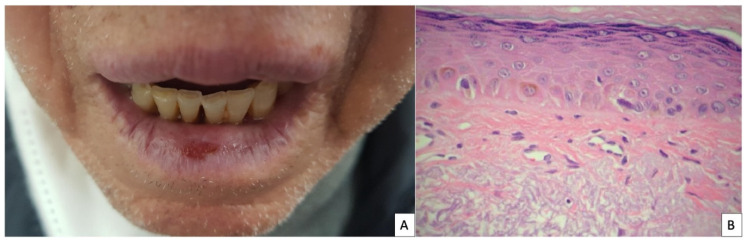
(**A**) Clinical image: poorly demarcated border of the lower lip with a erosion (**B**) Histopathological image, adapted with permission from Ref. [12] Copyright 2012 Vieira et al.: hyperkeratosis and atrophy of the epidermis, with discrete dysplasia, and elastosis of the dermal collagen (HE 200×).

Actinic cheilitis (AC) is a chronic inflammation of the lip, with exposure to ultraviolet radiation being the most important etiologic agent in its development. The prevalence of AC has been reported to vary between 0.45% and 2.4%, and increasing up to 43.2% if only outdoor workers are considered [1,2]. Risk factors for the development of AC are outdoor work, smoking, fair skin and immunosuppression. AC is clinically characterized by a poorly demarcated border of the lip with a white scaling plaque, erythema, atrophy, edema, hyperkeratosis and erosions (Figure 1A). The lower lip is the most frequently affected location, which is usually affected in a diffuse manner, although it can also present as a localized lesion in a small area [3]. Dermoscopy shows white structureless areas, vascular telangiectasia, scales and erosions [4]. Its diagnosis is usually based on presenting history and clinical evaluation, although a lip biopsy is mandatory in case of infiltrating lesions suggestive of squamous cell carcinoma (SCC). The most common histopathological findings are epithelial dysplasia (singularly cell atypia and loss of polarity of keratinocytes) jointly with hyperkeratosis, parakeratosis, acanthosis, solar elastosis and inflammatory infiltrates (Figure 1B) [5].

AC is considered an oral potentially malignant disorder (OPMD) that is associated with a statistically increased risk of developing SCC [6]. A recent systematic review of the literature reported a malignant transformation rate of 3.07% [7]. In addition, a significant percentage of the lower lip carcinomas are linked to pre-existing AC lesions [8]. The rate of nodal metastasis of lip SCC is four times higher than it is for cutaneous SCC [9], and the overall 5-year survival rate reported is 79% [10]. Lip SCC is a relevant public health problem, with a rate of new cases of 0.6 per 100,000 per year, and a death rate of 0.02 per 100,000 annually, as communicated in previous official reports, such as the SEER program (Surveillance, Epidemiology, and End Results cancer registry, National Cancer Institute of the United States of America) [11]. These data support the importance of early detection and treatment of AC to prevent the development of SCC.

The treatment of AC is difficult due to its anatomic location (i.e., proximity of the mucosa and cosmetically sensitive area). Moreover, there is no consensus regarding the optimal treatment approach. Surgical vermilionectomy offers a definitive treatment, but it has significant adverse effects such as scarring and altered sensitivity or functionality of the lip [13]. Other physical treatments include cryosurgery, electrodessication or laser therapy. In addition, photodynamic therapy (PDT) for AC has been reported in a number of studies with different results. Topical treatments include chemo or immunotherapy such as 5-fluorouracil (5-FU), imiquimod, diclofenac, trichloroacetic acid or ingenol mebutate. These are safer long-term treatments, but local adverse effects during treatment application, such as edema, erythema or erosions, reduce patient adherence, together with the disadvantage of having shown worse efficacy in terms of recurrence [14]. Laser therapy is a treatment option less invasive than surgery, with lower adverse events (AEs) and similar efficacy. But evidence on the efficacy relies on several primary-level studies, and high-quality evidence is still lacking.

**Figure 2:**
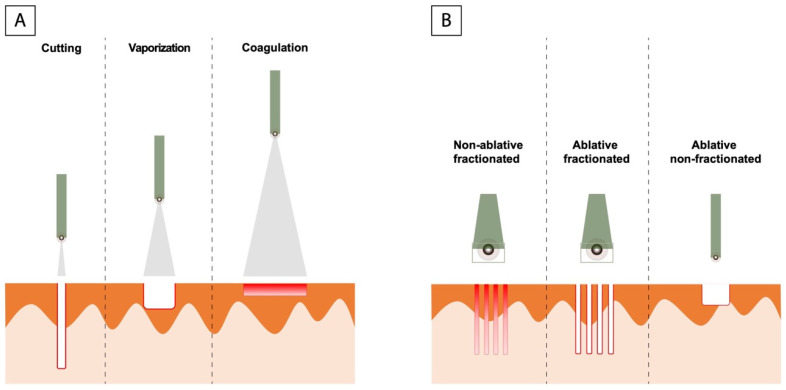
(**A**) Focused light with a very small beam of incidence on the skin (0.1–1 mm) causing pinpoint injury, which allows us to use it as a cutting system for cutting. Separating the laser handpiece from skin we produce a larger spot (2–5 mm), increasing the area of laser-tissue interaction, and thus vaporizes the tissues. Finally, a large spot size (4 mm) causes a drop in irradiance, resulting in non-ablative coagulation, which allow hemostasis of small bleeding vessels. (**B**) Types of CO_2_ laser: Non fractionated laser acts on the entire treated area, however fractionated lasers treat only small columns of the treated skin, known as microthermal zones (MTZs). These MTZs can be non-ablative dermal injuries or both epidermal and dermal injuries in case of ablative fractionated laser.

Among different laser types, the carbon dioxide (CO_2_) laser is one of the most widely used in dermatology. Indications include treatment of benign or malignant lesions and the field of esthetic dermatology [15]. The CO_2_ laser emits light energy in the form of a beam of photons at 10,600 nm, which is absorbed by water, the specific chromophore. As a consequence, vaporization of the intracellular water produces an ablation of the tissue locally and a small amount of diffusion of thermal energy to adjacent structures, reaching a good hemostasis. Through moving the handpiece towards or away from the focal point of the beam, a CO_2_ laser can be used for cutting, vaporization or coagulation [16]. There are different types of CO_2_ lasers: fractionated (ablative and non-ablative) and non-fractionated (Figure 2). In addition, this type of laser can be used in two different modes: continuous-wave (a continuous beam of light without power variation) or pulsed. Finally, there are also the super-pulsed and ultra-pulsed mode that have allowed treatment with maximum power and very short pulse durations (0.1–100 milliseconds, several hundred pulses per second), achieving high peak power to cut tissue precisely and a minimization of energy diffusion to adjacent tissue effects. Erythema and oedema of the area are the most frequent side effects after treatment, in addition to pain associated with the procedure or post-treatment pain.

Based on this background, we have carried out the first systematic review with the aim of analyzing the degree of evidence of laser for the treatment of AC in terms of efficacy and safety. In addition, we evaluated the core outcome set for actinic keratoses recently defined, adapted to AC [17]: complete clearance, severity of AEs, patient perspective on effectiveness, patient-reported future treatment preference and recurrence rate.

## 2. Materials and Methods

The present systematic review was conducted in accordance with Cochrane Collaboration criteria [18] and closely adhered to the Preferred Reporting Items for Systematic Reviews and Meta-Analyses (PRISMA) reporting guidelines [19] (Supplementary File Table S1). Under the premise of minimizing the risk of bias, a protocol containing the methodology followed in this systematic review was designed prior to initiation and registered in the PROSPERO international prospective register of systematic reviews (ID313274).

### 2.1. PICO Question

To assess the degree of evidence of laser therapy for the treatment of AC, the PICO framework was designed: Population: Participants with AC diagnosed by clinical and/or histopathological criteria; Intervention: laser treatment; Comparison: other therapeutic approaches; Outcome: efficacy and safety.

### 2.2. Search Strategy

A broad search was performed in MEDLINE (though PubMed), Embase, Scopus, Web of Science and Cochrane Central Register of Controlled Trials (CENTRAL) electronic databases. The search strategy was elaborated by combining thesaurus terms with free terms (Supplementary File Table S2). No limits were applied with regard to year or language of publications. A manual search was conducted in the reference lists of all selected studies for additional relevant publications. This search was conducted in June 2021 and updated in January 2022. All records were processed using a bibliographic reference manager software (Mendeley v.1.19.8, Elsevier, Amsterdam, The Netherlands).

### 2.3. Selection Criteria

The following inclusion criteria were taken into consideration:

- longitudinal studies (clinical trials, prospective or retrospective studies, case series and case reports) in which the laser treatment of AC was evaluated;
- at least one follow-up visit;
- studies reporting clinical response as outcome.

Exclusion criteria include:

- in vitro or in vivo animal studies;
- observational cross-sectional studies with no follow-up visits;
- reviews or meta-analyses, personal opinions or comments;
- not reported outcomes.

### 2.4. Study Selection and Data Extraction

Studies were selected on the basis of precedent inclusion and exclusion criteria by two blinded reviewers (AAR and RRV). Discrepancies were resolved by consensus. The selection of the studies was carried out in two independent phases, the first according to the titles and abstracts of the articles, and the second in which a complete reading of articles selected in the previous phase was conducted.

Data extraction from the selected articles was carried out independently by two authors (AAR and RRV), using a standardized data extraction form. The following information was gathered: first author, publication date, study design, continent and country, follow up period, number of patients enrolled, intervention and comparison, modality of laser, treatment protocol, sex and age of patients, location of AC, percentage of affected lip, risk factors, previous treatment. Outcomes analyzed were clinical clearance, recurrence rate, malignant transformation rate, cosmetic results, histological findings, AEs, and any other information deemed necessary to assess methodological quality.

### 2.5. Risk of Bias Assessment

The methodological quality across primary-level studies was critically appraised using the *Cochrane Collaboration’s RoB-2 tool for assessing risk of bias* [20]. The following potential biases were explored categorized into five specific domains: (1) randomization process; (2) deviations from intended interventions; (3) missing outcome date; (5) selection of the reported result. The risk of potential bias was evaluated as low risk of bias, some concerns, or high risk of bias for each domain. These domains were independently evaluated in each primary-level study, recording the particularities and potential biases observed. A sixth and final domain assesses the overall risk of bias based on the previous five domains. The risk of bias plots were drawn using the Cochrane robvis web app [21].

### 2.6. Synthesis of Results

Data were analyzed through narrative synthesis methods, allowing the analysis of qualitative and quantitative data extracted from primary-level studies. For each study, the mean age of all patients and the relative frequencies of the main outcomes were calculated with Microsoft Excel 2010 (Microsoft Corp., Redmond, WA, USA). Data are presented in tables, sorted according to the publication year of each study jointly with their more relevant methodological and clinical features. Meta-analysis was not performed due to the expected methodological and clinical inter-study heterogeneity, mainly attributed to differences related to study design or intervention components (see protocol). Subgroup analysis based on study types (RCTs vs. non randomized studies) has not been carried out due to the limited number of RCTs conducted in this topic yet.

## 3. Results

### 3.1. Study Selection

**Figure 3:**
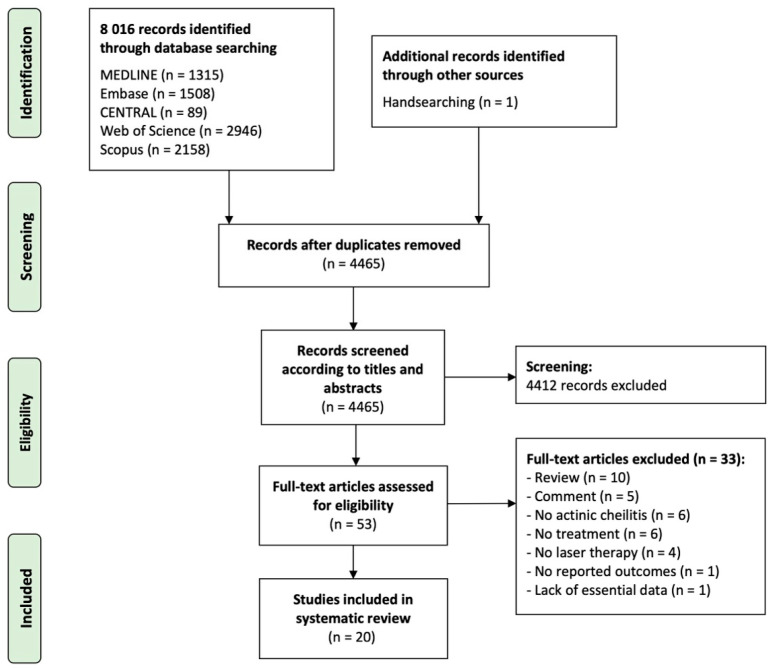
Flow diagram showing the identification and selection process of primary-level studies in this systematic review, analyzing the clinical implications of laser therapy in patients suffering from actinic cheilitis.

Figure 3 shows the flow diagram with the study identification and selection process. The search strategy across databases retrieved a total of 8016 articles: 1315 from PubMed, 1508 from Embase, 89 from CENTRAL, 2946 from the Web of Science, 2158 from Scopus and one additional article identified after handsearching a reference list from selected articles. After duplicates were removed, 4465 articles were identified and screened, reviewing titles and abstracts, so that 4412 were subsequently excluded as they did not meet eligibility criteria. The full text of the remaining 53 articles was systematically reviewed, and after exclusion criteria were applied, 33 articles were excluded (Supplementary File Table S3 list studies excluded and their exclusion reasons). Lastly, 20 studies were included in the final sample and detailed data extraction and data analyses was performed on them [22,23,24,25,26,27,28,29,30,31,32,33,34,35,36,37,38,39,40,41].

### 3.2. Study Characteristics

A total of 20 primary-level studies were included in this systematic review, published between 1985 and 2015. The characteristics of the selected studies are summarized in Table 1. Four of the studies were randomized clinical trials (RCTs), nine prospective case series, six retrospective case series and one case report. Eleven studies were conducted in North America, four in Europa, three in Asia and one in South America and Oceania, respectively. The total number of patients included was 512, of which 463 were treated with laser and 103 received other types of therapy as control arm (a total of 54 patients received split-mouth treatment, receiving two treatments in different halves of the lip). Sample sizes in each simple study ranged from 1 to 99 patients.

Three different types of lasers were used in the 20 studies: CO_2_, Er:YAG and 1927-nm thulium laser. The most studied laser therapy modality was CO_2_ laser, evaluated in 15 of the 20 studies (14 laser ablation, 1 laser vermilionectomy) (348 patients), one of them compared two different methods of CO_2_ laser (40 patients), one comparing it with electrodessication (14 patients) and another paper with 3 different treatment arms (topical 5-FU, chemical peel, vermilionectomy) (40 patients). Regarding the parameters employed for the CO_2_ laser treatment, 13 studies used the continuous wave mode versus 3 that used the pulsed mode, with power ranging from 2 to 80 W (this in the case of super-pulsed mode), and irradiance between 100–300 W/cm^2^. Two studies examined a combination of laser ablation with PDT (54 patients), one using long-pulsed pulsed dye laser and another one Er:YAG ablative fractional laser, two studies evaluated Er:YAG laser ablation (111 patients), with spots of 3–4 mm and irradiance between 1 to 24.05 J/cm^2^, and one study examined a 1927-nm thulium laser (1 patient) with an irradiance of 20 mJ/cm^2^.

### 3.3. Patients’ Characteristics

Information on gender was recorded for 423 patients (16 studies). Table 2 summarizes the demographic and clinical characteristics of the recruited patients. The majority of patients were male, 79.67% (*n* = 337) vs. 20.33% female (*n* = 86); the gender of 89 patients was not reported. The age of patients ranged from 26 to 92 years, with a mean age of 59.92 ± 7.68 (15 studies). The location of AC was reported for a total of 216 patients (11 studies), among which 93.52% (*n* = 202) had AC on the lower lip, 0.46% (*n* = 1) on the upper lip and 6.02% (*n* = 13) on both lips. The percentage of lip involvement was only communicated in 94 patients (5 studies): 66 patients had more than 50% surface area involvement, 14 patients more than 75% and 14 patients more than 85%.

Information on risk factors for AC development was gathered from 13 studies (331 patients), although not all studies specifically report the number of patients with each risk factor described. The most frequent risk factor was fair skin, present in 191 patients. In addition, 100 patients were smokers and 25 occasionally consumed alcohol, 100 were outdoor workers, 14 reported intense sunlight exposure, 46 had a personal history of NMSC and 7 had a history of SCC of the lower lip. Finally, 12 studies reported whether patients had previously received treatment and what type of treatment. Of a total of 234 patients, 47.44% (111 patients) had previously received treatment. Cryotherapy was the most commonly used therapy, being applied in 59 patients, 16 patients were treated with surgery (vermellectomy), 12 with 5-FU, 6 CO_2_ laser, 5 electrodesiccation, 3 topical retinoids, 2 topical steroids and 1 imiquimod. The method used was not specified in 14 patients (it should be noted that several patients had received more than one treatment type).

### 3.4. Risk of Bias Analysis

**Figure 4:**
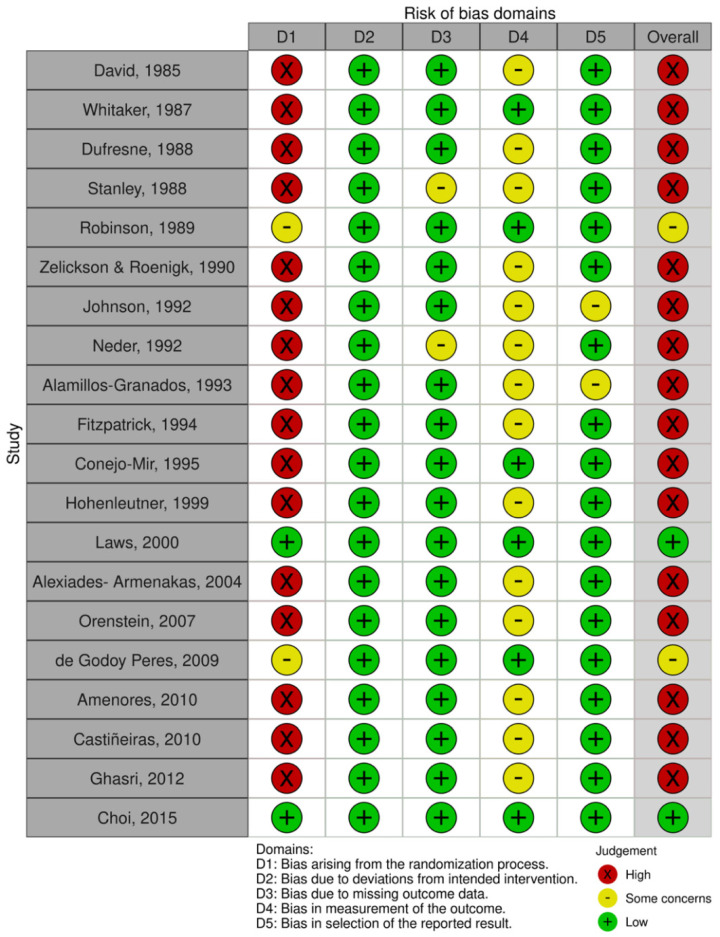
Quality plot graphically depicting the critical appraisal categorized in bias domains, across primary-level studies, using the Cochrane Collaboration’s tool for assessing risk of bias (RoB-2 tool). Green, low risk of potential bias; yellow, some concerns; red, high risk of potential bias.

**Figure 5:**
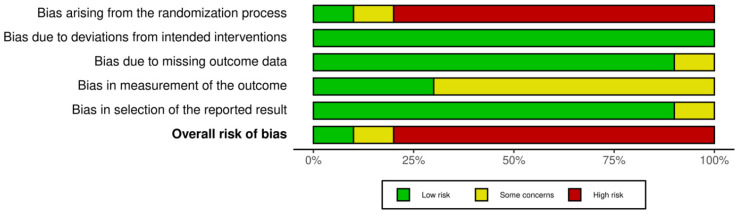
Horizontal bar plot showing the quantification of the risk of potential bias across studies for each risk of bias domain, expressed as percentages, assessed with the Cochrane Collaboration’s tool for assessing risk of bias (RoB-2 tool). Green, low risk of potential bias; yellow, some concerns; red, high risk of potential bias.

Figure 4 and Figure 5 show risk of bias summary and the graph resulted after evaluating the studies included in this review. Eighty percent of studies were judged at a high risk of bias, ten percent at a low risk of bias and the other ten percent at some concerns. Most of the studies were non-randomized observational cohort studies, so the randomization process significantly affected the quality rating of studies. Four studies performed patients’ randomization, two of them described the method used to conceal the allocation sequence. Only one of the studies reported blinding for treatment (not possible in participants due to the physical nature of the type of therapy). Bias from the measurements of the outcomes aroused some concerns in nearly 75% of studies. Finally bias due to deviations from the intended intervention or bias in the selection of the report result were minimal or not detected.

### 3.5. Clinical and Histopathological Outcomes

Table 3 summarizes the clinical and histopathological outcomes systematically reviewed. The main outcome to assess the efficacy was complete clinical clearance of AC, present in a rate of 92.5% patients, 100% if only studies evaluating Er:YAG laser are considered. Fifteen studies had a complete clearance in all patients (288 patients), while in the other five studies it did not occur in 18 patients out of a total of 174. The worst results were reported in a study that evaluated the combination of laser with PDT, showing complete clearance in 68.42% and partial clearing in 10.53%, with a failure to follow-up occurring in 15.79%.

Eighteen studies evaluated the recurrence rate and malignant transformation to SCC. Eight studies had no recurrences, and of the ten studies that showed it, the range was 5.26 to 21.43%. The studies examining laser therapy in combination with PDT had recurrence rates of 8% (vs. 50% of PDT alone) and 5.26%. Malignant transformation after laser therapy was examined in the same eighteen studies. Only three studies observed cases of post-treatment malignant transformation, with low rates: 2.33% (1/43), 4.65% (2/43) and 7.14% (1/14).

In terms of the histopathologic outcomes, a post-treatment biopsy was performed in 5 studies, showing histological improvement in terms of dysplasia in all biopsies in 4 of the 5 studies, while in the remaining study only 40% of the biopsies performed showed improvement. On the other hand, healing time was estimated in 12 studies, all of them showing a similar range of time, from 14 to 49 days, reduced to 28–49 days if laser vermilionectomy is not considered.

Fourteen articles (331 patients) assessed the cosmetic result after treatment, although in most of them it was reported as aggregate data. Cosmetic outcome was described as excellent in 9 studies, optimal in 4 and good in 1. Three studies reported individual data: Choi et al. reported excellent or good results in 73% of cases, while fair or poor outcomes were observed in 27% of cases; Amenores et al. described improved cosmetic result in 92.2% of patients, no change in 6.5% and 1.3% mildly worse; and the last study observed an improved cosmetic result in 60.5%, no change in 37.2% and worsening in 2.3%.

All of the included studies reported AEs following treatment, in two of which there were none of the 17 patients included. No patient discontinued treatment due to these AEs. The most common side effects included postoperative symptoms: bleeding (0–53%), pain (0–100%), erythema (0–100%), edema (0–43%), and a burning sensation (0–100%), which generated difficulty eating in the immediate postoperative period. Moreover, two studies provided information regarding pain intensity: slight to mild in 86–93%, and moderate to severe in 7–14% of cases. Less frequent AEs were infection (6 patients) or herpes simplex reactivation (3 patients). Hypertrophic (5 patients) or non-hypertrophic (26 patients) scarring, dysesthesias (9 patients), worse lip function (1) or salivary gland cysts (5 patients) were the persistent AEs identified.

Patient satisfaction or acceptance was reported in only 6 studies, generally being a well-tolerated procedure with an excellent degree of patient satisfaction in 5 of the 6 studies (99 patients). Amenores et al. showed more precise information on the degree of patient satisfaction, such that 68.8% were very satisfied, 24.7% satisfied, 3.9% strongly disliked, 1.3% disliked and 1.3% neutral. In addition, 87% of patients would repeat laser therapy for AC again. In addition, no patient was reported to have discontinued treatment. No study reported information on patients’ perspectives on effectiveness.

## 4. Discussion

The present study is the first systematic review which attempted to evaluate the degree of evidence for laser therapy as a treatment for AC. Based on a sample of 20 primary level studies with a total of 512 recruited patients, laser therapy showed a complete clearance of AC in 92.5% of patients (100% if only studies evaluating Er:YAG laser are considered), with a maximum recurrence rate of 21.43%. The most important purpose of AC therapy is to minimize the risk of this potentially malignant lesion progressing to SCC. This systematic review shows the high capacity of laser therapy to achieve this objective, since it shows a very low frequency of malignant transformation after treatment (detected in only 3 of the 20 studies analyzed). In addition, cosmetic outcomes and patient satisfaction were described as excellent in most studies. Our results are superior to those reported in a systematic review of similar characteristics carried out to evaluate the effectiveness of PDT as a therapeutic option for AC [42], with a complete response in 139 of 233 subjects treated, and good to excellent cosmetic outcomes in the majority of subjects. Laser therapy also shows superior results to treatment with topical anti-inflammatory and antineoplastic agents. In another systematic review [43], 5-FU showed recurrences in 20–50% of patients, diclofenac had a complete clearance rate of 20–71%, and imiquimod and ingenol mebutate showed mixed results, the recurrence was 0% in both cases, however the clinical complete response rate was only 50% and 40%, respectively. Finally, although vermilionectomy may represent an advantage over laser therapy in the histological examination of the resected tissue, laser therapy has been associated with fewer AEs than surgery [36]. However, future primary-level studies should directly compare both approaches, mainly new surgical techniques such as W-plasty that may be able to provide the same outcomes as classic vermilionectomy with less scarring [44]. Laser therapy confers an additional benefit in patient adherence given its one-shot treatment nature, whereas topical therapies can be discontinued because the patient must be applying the treatment for several days, and the appearance of AEs may cause treatment interruption. However, there are no treatment protocols established regarding the physical parameters of the laser or the number of sessions required for a correct treatment, which complicates the reproducibility of the studies.

Few studies assessed histologic outcomes after treatment. Whitaker et al. observed not cellular atypia and a marked diminution of solar elastosis after CO_2_ laser ablation in 16 patients [23]. Similar results were observed in the 10 patients of the study by Robinson et al. [36] and in the 5 biopsied patients among the 25 in the Conejo-Mir et al. study [24]. In the article by De Godoy et al., reduced degrees of dysplasia with significant difference between preoperative and postoperative degrees of epithelial atypia were observed [29]. Finally, Laws et al. found that of the 5 patients who were biopsied (out of 14 patients), only 2 showed improvement [26]. In addition, an article performed biopsies during treatment with the aim to histologically characterize the tissue zones seen after laser impact and thereby determine the optimal depth of destruction of diseased tissue. Complete destruction of the epithelial layer was observed in all the specimens irrespective of the number of laser passes [38]. On the other hand, the longest healing time range was reported in the study by Alamillos-Granados et al. [40], in which CO_2_ laser vermilionectomy was performed instead of ablation, which lengthened the healing time to 28–49 days. If this study is excluded, the healing ranges of the other studies are reduced to 14–35 days. In addition, an article reported that the CO_2_ laser-treated side healed significantly faster than the side treated with electrodessication (*p* < 0.001) [26].

Most of the studies used CO_2_ laser as therapy; thus, our findings mainly refer to this modality. Our results support the efficacy of CO_2_ laser therapy and are consistent with the National Comprehensive Cancer Network Guidelines for Squamous Cell Skin Cancer that consider ablative laser to be a valuable therapeutic option for AC [45]. Nevertheless, given promising results shown in the two studies using Er:YAG laser, new studies should be conducted to validate the high efficacy of this treatment. Moreover, our systematic review is consistent with a narrative review of the literature that gave treatments grade A through D based on the strength of the evidence [13]. CO_2_ laser was given the highest grade (A–B), surgery was rated B-C due to invasiveness of the procedure, while trichloroacetic acid and chemical peels were given the lowest grade (D) recommendation. Other topical therapies (cryosurgery, 5-FU, imiquimod and PDT) were all given grade B.

There is no established clinical measurement tool for evaluating severity and treatment outcomes for AC. In the absence of such tools, this systematic review was conducted on the basis of a recent consensus that established international core outcomes set for clinical studies on the treatment of actinic keratosis (AKs) based on Delphi surveys of physicians and patients stakeholders [17]: complete clearance of AKs, percentage of AKs cleared, severity of AEs, patient perspective on effectiveness, patient-reported future treatment preference, and recurrence rate. The majority of our selected studies reported on the clearance of AC (not the percentage of cleared ACs as it is considered a single lesion), recurrence and malignant transformation rates, side effects, healing time and cosmetic outcome. However, few studies assessed patient satisfaction and none assessed patients’ perspectives of efficacy. The development of a similar consensus for AC would be beneficial, given the particularities of this lesion in terms of cosmetic results and functionality. In addition, patient satisfaction and effectiveness perspectives need to be closely examined in future studies.

According to our qualitative analysis using Cochrane Collaboration’s tool for assessing risk of bias, we also should point out that the primary-level studies included in this systematic review have not been conducted with the same methodological rigor. The domains related to randomization and blinding practices across research harbored the higher risk of potential bias. Blinding is a key research practice that should always be applied to avoid the introduction of systematic errors in almost every study design, from preclinical research, to observational or interventional studies. A study that pursues integrity and high methodological standards will obtain results with higher internal validity by an investigator blinded to the experimental versus control groups. New studies carefully designed are needed to offer a higher quality of evidence, which should consider the potential biases and recommendations reported in this systematic review, to improve and standardize future research.

Our systematic review also presents some potential limitations that should be discussed. First, as expected and stated in our study protocol, a considerable degree of clinical and methodological heterogeneity was encountered. Consequently, meta-analysis could not be performed (i.e., the statistical combination of results from separate primary-level studies) in order to obtain single pooled estimates. Very different study subpopulations were identified, which must imperatively be considered and managed as true sources of heterogeneity (e.g., differences among the wide range of experimental interventionist methods, such as type of laser -(1) CO_2_ laser, (2) Erbium:YAG laser, (3) 1927-nm thulium laser- or physical parameters; differences between target populations, singularly general populations vs. outdoor workers exposed to ultraviolet radiation; differences in terms of global solar ultraviolet index across geographical areas; differences in study design across primary-level studies, with different samples, heterogeneous comparison methods and research practices). Future studies are needed to report data from more homogeneous subgroups to allow the application of meta-analytic techniques to investigate more robustly the magnitude, precision and direction of the effect. Second, an inherent limitation of the included studies was the lack of reporting of relevant datasets that limited the number of observations for descriptive analysis and narrative synthesis of current evidence (e.g., influence of sex, age, tobacco, professional activity, lip subanatomical location, etc.). Future studies should report datasets in a more rigorous way—preferably through individual participant data—given the clinical and methodological relevance of these variables, needed for future adjusted, stratified and meta-regression analyses using meta-analytic techniques in upcoming studies. Despite the above limitations, study strengths include our careful study design; a comprehensive literature search strategy—not restricted to date or publication language—where more than 8000 registers were screened to identify a robust sample size (i.e., 20 primary-level studies) as a result of the efforts made to reduce the potential risk of selection bias, one of the Achilles’ heels of this study design; a robust qualitative analysis offering recommendations for the development and design of future studies on this topic; and a singular emphasis on the potential translational opportunities derived from our evidence synthesis analysis.

## 5. Conclusions

Our systematic review synthetizes and critically appraises the current available knowledge on laser therapy for AC and offers results based on the highest level of evidence provided to date, confirming high efficacy in terms of complete clearance and recurrence rate. However, larger blinded randomized controlled studies are necessary to validate these conclusions, and consequently to determine the optimal therapeutic strategy for AC patients. In addition, future studies should incorporate measures of patient satisfaction and preference to achieve an optimal approach to the management of this prevalent OPMD with direct translational potential for clinical practice.

## Figures and Tables

**Table 1:** Study characteristics.

Study	Study Design	Continent (Country)	Type of Treatment	Treatment Protocol *	No. Patients	No. Controls	Follow-Up
**David, 1985**	Retrospective case series	North America (USA)	CO_2_ laser ablation	CW, 15 W, 3 mm, 300 W/cm^2^ 1 session, 1–3 passes	8	0	34 (27–38 months)
**Whitaker, 1987**	Prospective case series	North America (USA)	CO_2_ laser ablation	CW, 4–8 W, 2 mm, 133–256 W/cm^2^ 1 session, 2 passes	16	0	≥24 months
**Dufresne, 1988**	Prospective case series	North America (USA)	CO_2_ laser ablation	CW, 3–5 W, 2 mm, 100–160 W/cm^2^ SP, 3–5 W, 200–300 μsec pulse width, 200 to 300 repetitions per second, 2 mm 1 session	13	0	11 (3–24 months)
**Stanley, 1988**	Prospective case series	North America (USA)	CO_2_ laser ablation	CW, 2–3 W, 1 mm 1 session, 1–3 passes	3	0	18 months
**Robinson, 1989**	RCT	North America (USA)	CO_2_ laser ablation	CW, 5 W, 1 mm, 200–250 W/cm^2^ 1 session, multiple passes	10	30 (topical 5-FU, chemical peel, vermilionectomy)	50 months
**Zelickson & Roenigk, 1990**	Prospective case series	North America (USA)	CO_2_ laser ablation	CW, 5–7 W, 2 mm, 1 session	43	0	20 (>10 months)
**Johnson, 1992**	Prospective case series	North America (USA)	CO_2_ laser ablation	CW, 2–3 W, 1 mm 1 session, 1–3 passes	14	0	12 months
**Neder, 1992**	Retrospective case series	Asia (Israel)	CO_2_ laser ablation	Repeated pulse, 8 W 1 session	16	0	12 months
**Alamillos-Granados, 1993**	Prospective case series	Europe (Spain)	CO_2_ laser vermilionectomy	CW, 8 W 1 session	19	0	NR
**Fitzpatrick, 1994**	Retrospective case series	North America (USA)	CO_2_ laser ablation	CW, 0.5–10 W, 3–4 mm SP, 80 W peak power, 200–400 ms, 2.5 mm 1 session, repeatedly passes	35 (17 CM, 18 SP)	0	8–30 months
**Conejo-Mir, 1995**	Prospective case series	Europa (Spain)	CO_2_ laser ablation	CW, 5 W, 2 mm, 100–160 W/cm^2^ 1 session, 1–3 passes	25	0	36 months
**Hohenleutner, 1999**	Prospective case series	Europa (Germany)	CO_2_ laser ablation	CW, 10 W 1 session, repeatedly passes	19	0	16 (2–60 months)
**Laws, 2000**	RCT	North America (USA)	CO_2_ laser ablation vs. electrodessication	Short pulse 18 W, 360 mJ/cm^2^, 7 × 3.5 mm rectangular pattern 1 session	14	14	3 months
**Alexiades-Armenakas, 2004**	Prospective case series	North America (USA)	Laser mediated LP PDT vs. LP PDT alone	75 J/cm^2^, 10 ms, 10 mm 1–3 sessions, 1–3 passes	19	2	12 months
**Orenstein, 2007**	Retrospective case series	Asia (Israel)	Er:YAG laser ablation	3 mm, 16.97–24.05 J/cm^2^, 1 session, repeatedly passes	12	0	23.16 (8–36 months)
**de Godoy** **Peres, 2009**	RCT	South America (Brazil)	CO_2_ laser ablation (350 vs. 250 mJ)	CW, 350 mJ, 3.5 W, amplitude of 0.1 s, repetition rate of 6.6 Hz CW, 250 mJ, 5 W, amplitude of 0.05 s, repetition rate of 10 Hz 1 session	40	40	19.45 (6–30 months)
**Amenores 2010**	Retrospective case series	Oceania (Australia)	Er:YAG laser ablation	4 mm, 1 J/cm^2^, 2–35 or 400 ms. 1 session, 1–4 passes	99	0	65.7 (10.0–99.2 months)
**Castiñeiras, 2010**	Retrospective case series	Europe (Spain)	CO_2_ laser ablation	CW, 2 W/cm^2^ 1 or more sessions, several passes	43	0	29.4 (13–60 months)
**Ghasri, 2012**	Case report	North America (USA)	1927-nm thulium laser	20 mJ/cm^2^ 3 sessions, 4 passes	1	0	Not reported
**Choi, 2015**	RCT	Asia (South Korea)	Er:YAG ablative fractional laser + MAL-PDT vs. MAL-PDT	300 μm ablation depth, single pulse 1 session	14	19 (MAL-PDT)	>12 months

* Parameters of laser: CW = continuous wave, SP = superpulsed, W = watts of power, diameter of spot size, irradiance. Abbreviations: RCT, randomized clinical trial; CO_2_, carbon dioxide; LP, (long-pulsed pulsed dye laser); PDT, photodynamic therapy; mJ, millijoule; Er:YAG, Erbium:YAG; MAL, methyl aminolevulinate; cm^2^, square centimeters; mm, millimeters; μsec, microsecond; 5-FU, 5-fluorouracil.

**Table 2:** Clinical and histopathological data of patients.

Study	No. Patients	No. Controls	Gender (M/F)	Mean Age (Range)	Location of AC (Lower/Upper Lip)	Percentage of the Lip Surface	Risk Factors for AC Development	Previous Treatment
**David, 1985**	8	0	6/2	56.6 (37–70)	8/2	NR	Fair skin (8)	8 (cryosurgery with liquid nitrogen, topical fluorouracil, electrodesiccation and curettage, or scalpel excision of local lesions).
**Whitaker, 1987**	16	0	15/1	NR (48–84)	16/0	>50%	NR	6 (4 cryosurgery, 2 electrodesiccation)
**Dufresne, 1988**	13	0	8/5	67	13/0	NR	SCC lower lip (3)	8 (2 cryosurgery, 6 5-FU)
**Stanley, 1988**	3	0	3/0	64 (46–78)	3/0	NR	Outdoor worker (2), Fair skin (1)	1 (topical not specificized)
**Robinson, 1989**	10	30	38/2	60.5 (51–70) vs. 62 (48–87)	10/10	>50%	Smoking (7/10, 24/30), NMSC (40/40), Fair skin (40/40)	0
**Zelickson & Roenigk, 1990**	43	0	38/5	70 (46–85)	43/1	NR	NR	NR
**Johnson, 1992**	14	0	12/2	63.5 (35–92)	14/0	>80%	Fair skin (14), history of long- term sun exposure (14)	0
**Neder, 1992**	16	0	NR	NR	NR	NR	NR	NR
**Alamillos-Granados, 1993**	19	0	19/0	61 (40–74)	NR	NR	Outdoor worker (12), SCC lower lip (3)	NR
**Fitzpatrick, 1994**	35	0	NR	NR	NR	NR	NR	NR
**Conejo-Mir, 1995**	25	0	19/6	54 (NR)	NR	NR	Outdoor worker, NMSC on the face (5)	7 (Cryosurgery 5, electrodesiccation 2)
**Hohenleutner, 1999**	19	0	NR	NR	NR	NR	NR	NR
**Laws, 2000**	14	14	13/1	NR (54–82)	14/0	>75%	NMSC of H&N, SCC lower lip (1)	0
**Alexiades- Armenakas, 2004**	19	0	NR	59 (45–75)	NR	NR	NR	15 (cryosurgery 12, 5-FU 12, CO_2_ laser 2, vermilionectomy 1)
**Orenstein, 2007**	12	0	7/5	52.67 (37–71)	12/0	NR	Smoking (6)	5 (vermilionectomy, 5-FU and electrosurgery)
**de Godoy** **Peres, 2009**	40	0	36/4	42.47 (26–76)	40/0	>50%	Fair skin (27) smoking (17), consumption of alcohol (25)	NR
**Amenores. 2010**	99	0	68/31	52.8 (28–85)	NR	NR	Smoking (46), outdoor worker (54), fair skin (57)	61 (cryosurgery 36, vermilionectomy 15, CO_2_ laser 4, retinoic acid 3, topical steroids 2, imiquimod 1)
**Castiñeiras, 2010**	43	0	34/9	70.54 (40–89)	42/1	NR	Fair skin (43)	NR
**Ghasri, 2012**	1	0	1/0	56	NR	NR	Fair skin (1), NMSC (1)	0
**Choi, 2015**	14	19 (MAL-PDT)	9/5 (11/8)	66.7 (49–84) vs. 69.4 (56–83)	NR	NR	NR	NR

Abbreviations: M, male; F, female; NR, Not reported; AC, actinic cheilitis; SCC, squamous cell carcinoma; NMSC, non-melanoma skin cancer; H&N, Head and neck; 5-FU, 5-fluorouracil; CO_2_, carbon dioxide.

**Table 3:** Clinical and histopathological data of patients.

Study (Patients/Controls)	Healing Time (Days)	Complete Clearance of AC	Recurrence Rate N (%)	Malignant Transformation N (%)	Cosmetic Result	Follow-Up Biopsy	Adverse Effects	Patient satisfaction Acceptance	Patient Perspective on Effectiveness	Treatment Discontinuation
**David, 1985** **(8)**	14–21	Yes	0	0	Excellent (no scarring)	NR	a band of fibrous tissue (1)	Excellent	NR	0
**Whitaker, 1987** **(16)**	14–28	Yes	1 (6.25)	0	Excellent (no scarring or changes in oral commissure)	Yes (not cellular atypia, a marked diminution of solar elastosis)	None	Excellent	NR	0
**Dufresne, 1988** **(13)**	21–28	Yes	0	0	Optimal (minimal scarring)	NR	Pain (2), Postoperative infection (2), focal lineal scarring (2), hypertrophic scar (1) hyperesthesia (1)	Procedure well tolerated	NR	0
**Stanley, 1988** **(3)**	NR	No (one patient residual AC after 6 weeks, a second passe was necessary)	0	0	Excellent (no scarring or functional disorders)	NR	Discomfort and edema (1)	NR	NR	0
**Robinson, 1989** **(10/30)**	14–30	Yes	0 5 (50) 5-FU, 7 (70) chemical peel	0	NR	Yes (not cellular atypia)	Difficulty eating in postoperative period (10/10)	NR	NR	0
**Zelickson & Roenigk, 1990** **(43)**	21–28	Yes	3 (6.98)	1 (2.33)	Optimal (26 improve, 16 unchanged, 1 worse)	NR	Scars after biopsy (3), mild postoperative pain (3), worse lip function (1)	NR	NR	0
**Johnson, 1992** **(14)**	14–28	Yes	0	0	NR	NR	Minimal (12), moderate (1) or severe (1) postoperative pain, hypertrophic scarring that resolved spontaneously (1).	NR	NR	0
**Neder, 1992** **(16)**	NR	Yes	NR	NR	Excellent	NR	Minimal pain	NR	NR	0
**Alamillos-Granados, 1993** **(19)**	28–49	Yes	NR	NR	Good	NR	Pain (7), edema (5), bleeding (2), fibrous band (3)	Excellent	NR	0
**Fitzpatrick, 1994** **(35)**	14–35	Yes	0	0	NR	NR	Adverse Healing (8 CW/3 SP), hypertrophic scarring (3 CW/0 SP), nonhypertrophic scarring (4 CW/1 SP), complaining of tightness of the lips without visible scarring (4 CW/1 SP)	NR	NR	0
**Conejo-Mir, 1995** **(25)**	21–30	Yes	0	0	Excellent (no scarring visible or functional disorders)	5 patients (no features of histological recurrence)	Minimal bleeding during the first 4–7 days (3). Slight paresthesia (1), scar contraction slight (1) and marked (1)	NR	NR	0
**Hohenleutner, 1999** **(19)**	NR	Yes	1 (5.26)	0	Optimal	NR	Little plane scar (2), palpable scar less than 5 mm in diameter (1)	NR	NR	0
**Laws, 2000** **(14/14)**	7–23 vs. 11–37	Yes	3 (21.43)	1 (7.14)	NR	5 patients (2 with residual AC)	Minimum pain (14), burning sensation (12)	NR	NR	0
**Alexiades-Armenakas, 2004** **(19/2)**	NR	No (Improvement in all patients, and complete clearing in 68%, partial in 10.53%)	1 (5.26)	0	NR	NR	Pain (8 slight, 5 mild, 1 moderate), erythema (2 slight, 10 mild, 4 moderate), impetigo (3)	NR	NR	0
**Orenstein, 2007** **(12)**	7–30	Yes	0	0	Excellent (no scarring visible or functional disorders)	NR	Pain (4), bleeding (5), paresthesia (4), edema (7), pyogenic granuloma (1), infection (1), slight tingling sensation (1)	NR	NR	0
**de Godoy** **Peres, 2009** **(40)**	10–22 vs. 12–24	No (general improvement in all patients, complete clearing in 88.5% for each protocol)	4 (12.5)	0	NR	26 patients (significant difference between pre and postoperative degrees of epithelial atypia for both protocols)	Immediate postoperative pain (12), HV-S reactivation (1)	NR	NR	0
**Amenores. 2010 (99)**	NR	No (general improvement in all patients, complete clearing in 95%)	15 (15.2)	0	Excellent (Improved in 92.2%, no change in 6.5% and 1.3% mildly worse; function not worsened)	NR	Postoperative/persisting symptoms: Pain (58/1), bleeding (53), swelling (43), exudation (42), erythema (37), burning (14/1), cracking (14/1), dryness (10/1), dysesthesias (8/2), pruritus (4/1). Scarring alterations (5), HV-S reactivation (2), salivary gland cysts (5)	High degree: 53 (68.8%) very satisfied, 19 (24.7%) satisfied, 3 (3.9%) strongly disliked, 1 (1.3%) disliked, 1 (1.3%) neutral. 67 (87%) would repeat it again	NR	0
**Castiñeiras, 2010** **(43)**	NR	Yes	3 (6.98)	2 (4.65)	Excellent (not contract scars or no function disorders)	NR	Minimal residual scar (2), not sensitive alterations	Excellent	NR	0
**Ghasri, 2012** **(1)**	NR	Yes	0	0	Excellent (not bruising or scarring)	NR	None	NR	NR	0
**Choi, 2015** **(14/19)**	NR	No (complete response at 3 months 92.31% (12/13) vs. 58.82% (10/17))	8 (1/12) vs. 50 (5/10)	0	Optimal (excellent or good in 73%, fair or poor in 27%)	Yes	Erythema (30), burning (30), swelling (5 vs. 6), hemorrhagic crusting (3 vs. 2), blistering (2 vs. 1)	NR	NR	0

Abbreviations: NR, Not report; AC, actinic cheilitis; 5-FU, 5-flurouracil; CW, continuous wave; SP, super pulsed; AC, actinic cheilitis.

## Data Availability

The data that supports the findings of this study are available in the supplementary material of this article.

## Supplementary Materials

The following supporting information can be downloaded at: https://www.mdpi.com/article/10.3390/ijerph19084593/s1, Supplementary File Table S1. PRISMA checklist; Supplementary File Table S2. Search strategy; Supplementary File Table S3. List of excluded articles with reasons.
