# Supplementary material for: Laser Therapy for the Treatment of Actinic Cheilitis: A Systematic Review
Source: Int J Environ Res Public Health. 2022 Apr 11;19(8):4593. doi: 10.3390/ijerph19084593 (PMC9028420; doi:10.3390/ijerph19084593)
Supplement: Supplementary file 1 [file ijerph-19-04593-s001.zip › Supplementary File Table S2.pdf]

Supplementary File Table S2. Search strategy.

Database	Query/Search Strategy	Items founds/Results	Search time limits
MEDLINE	“actinic cheilitis”[nm] OR "Cheilitis"[mh] OR ("Keratosis, Actinic"[mh] AND "Lip"[mh]) OR ((“lip”[all] OR “cheilitis”[all] OR “cheilosis”[all]) AND (actin*[all] OR “solar”[all] OR kerat*[all] OR precancer*[all] OR "potentially malignant"[all])) AND ("Therapeutics"[mh] OR “therapy”[sh] OR “treatment”[all] OR "Laser Therapy"[mh] OR laser*[all])	1,315	January, 2022
Embase	'actinic cheilitis'/exp OR ('actinic keratosis'/exp AND 'lip'/exp) OR (('lip' OR 'cheilitis' OR 'cheilosis') AND ('actin*' OR 'solar' OR 'kerat*' OR precancer* OR 'potentially malignant')) AND ('therapy'/exp OR 'treatment' OR 'laser therapy'/exp OR 'laser*')	1,508	January, 2022
CENTRAL	Title-Abstract-Keyword((lip OR cheilitis OR cheilosis) AND (actin* OR solar OR kerat* OR precancer* OR "potentially malignant")) AND (therap* OR treatment OR laser*)	89	January, 2022
Web of Science	TS=((lip OR cheilitis OR cheilosis) AND (actin* OR solar OR kerat* OR precancer* OR “potentially malignant”)) AND TS=(therap* OR treatment OR laser*)	2,946	January, 2022
Scopus	TITLE-ABS-KEY((lip OR cheilitis OR cheilosis) AND (actin* OR solar OR kerat* OR precancer* OR "potentially malignant")) AND (therap* OR treatment OR laser*)	2,158	January, 2022
Total	8,016		
