# Supplementary material for: Laser Therapy for the Treatment of Actinic Cheilitis: A Systematic Review
Source: Int J Environ Res Public Health. 2022 Apr 11;19(8):4593. doi: 10.3390/ijerph19084593 (PMC9028420; doi:10.3390/ijerph19084593)
Supplement: Supplementary file 1 [file ijerph-19-04593-s001.zip › Supplementary File Table S3.pdf]

**Supplementary File Table S3. List of excluded articles with reasons.**

| Author | Year | Title | Exclusion criteria applied |
|---|---|---|---|
| Horch, H H | 1982 | $CO_2$ laser treatment of oral dysplastic precancerous lesions: a preliminary report | No actinic cheilitis |
| MacFarlane, G | 1982 | Actinic cheilitis. Diagnosis, prevention, and treatment | Review |
| Horch, H H | 1986 | $CO_2$ laser surgery of oral premalignant lesions | No actinic cheilitis |
| Ries, W R | 1988 | Carbon dioxide laser treatment of actinic cheilitis | Review |
| Frankel, D H | 1989 | Carbon dioxide laser vermilionectomy for chronic actinic cheilitis | Review |
| Scheinberg, R S | 1992 | Carbon dioxide laser treatment of actinic cheilitis | Review |
| Sexton, J | 1993 | Carbon dioxide laser treatment for actinic cheilitis | Comment |
| Terezhalmy, G T | 1993 | Actinic cheilitis | Review |
| Vinciullo, C | 1996 | Combination carbon dioxide laser vermilionectomy and wedge excision of the lip | No actinic cheilitis |
| Dufresne, R G | 1997 | Actinic cheilitis. A treatment review | Review |
| Roberts 3rd, T L | 1997 | Aesthetic CO(2) laser surgery: evaluation of 907 patients | Lack of essential data |
| Wright, K | 1998 | Actinic cheilitis | No laser therapy |
| Kaugars, G E | 1999 | Actinic cheilitis: a review of 152 cases | No treatment |
| Gooris, P J | 1999 | Carbon dioxide laser evaporation of leukoplakia of the lower lip: a retrospective evaluation | No actinic cheilitis |
| Breuninger, H | 2000 | [CO2 laser vaporization in cheilitis actinica. Current comment on the contribution by S. Hohenleutner, M. Landthaler and U. Hohenleutner] | Comment |
| Karrer, S | 2001 | Role of lasers and photodynamic therapy in the treatment of cutaneous malignancy | Review |

| Author | Year | Title | Exclusion criteria applied |
|---|---|---|---|
| Vega-Memije, M | 2002 | Actinic prurigo cheilitis: clinicopathologic analysis and therapeutic results in 116 cases | No actinic cheilitis |
| Markopoulos, A | 2004 | Actinic cheilitis: clinical and pathologic characteristics in 65 cases | No treatment |
| Alexiades-Armenakas, M. | 2007 | Aminolevulinic acid photodynamic therapy for actinic keratoses/actinic cheilitis/acne: vascular lasers | Review |
| Dufresne, R G | 2008 | Dermabrasion for actinic cheilitis | No laser therapy |
| Shah, A Y | 2010 | Actinic cheilitis: a treatment review | Review |
| Ntomouchtsis, A | 2010 | Benign lip lesions. A 10-year retrospective study | No treatment |
| Gulec, A T | 2010 | Lip and oral mucosal lesions in 100 renal transplant recipients | No treatment |
| Wood, N H | 2011 | Actinic cheilitis: a case report and a review of the literature | No laser therapy |
| Jadotte, Y T | 2012 | Solar cheilosis: an ominous precursor part II. Therapeutic perspectives | Review |
| Cohen, J L | 2013 | Erbium laser resurfacing for actinic cheilitis | No reported outcomes |
| Goldman, G D | 2015 | Treatment of Actinic Cheilitis | Comment |
| Lopes, M L | 2015 | Clinicopathological profile and management of 161 cases of actinic cheilitis | No treatment |
| Dinani, N E | 2015 | Ablative fractional laser assisted photodynamic therapy for the treatment of actinic cheilitis | Comment |
| Plaza, J A | 2016 | Actinic Prurigo Cheilitis: A Clinicopathologic Review of 75 Cases | No actinic cheilitis |
| Sayan, A | 2018 | Revisiting lip shave: a solution for disorders of the vermilion border | No laser therapy |
| Brignardello-Petersen, R | 2019 | Evidence regarding how surgical treatment of actinic cheilitis compares with nonsurgical treatment is not trustworthy and does not consider all important outcomes | Comment |
| Gonzaga, A K G | 2020 | Actinic cheilitis: Morphometric parameters and its relationship with the degree of epithelial dysplasia | No treatment |
